# Review of the Urinary Schistosomiasis Control in Morocco (1960–2018)

**DOI:** 10.1155/2020/3868970

**Published:** 2020-10-14

**Authors:** A. Balahbib, F. Amarir, S. Bouhout, M. Rhajaoui, E. Adlaoui, A. Sadak

**Affiliations:** ^1^Laboratory of Biodiversity, Ecology and Genome, Faculty of Sciences, Mohammed V University, Rabat 10106, Morocco; ^2^National Reference Laboratory of Schistosomiasis and Malacology, Parasitology Department, National Institute of Hygiene, Agdal, Rabat, Morocco; ^3^Laboratory of Immunology and Biodiversity, Biology Department, Faculty of Sciences Ain Chock, University Hassan II, Casablanca, Morocco; ^4^Direction of Epidemiology and Disease Control (DELM), Ministry of Health, Rabat, Morocco

## Abstract

The purpose of this study is to describe the epidemiological profile and evolution of urinary schistosomiasis in Morocco, from the first confirmed case in 1960 until disease elimination, and control snails. During this period, 129,526 cases were recorded in Morocco. A majority of cases were reported in Agadir province (25%), Errachidia (18%), and Beni Mellal (13%). Other cases have been reported in the other provinces. Activities within the National Schistosomiasis Control Programme for more than three decades were focused in priori on screening in schools located in high-risk communities, treatment program, surveillance of snails in water bodies, and mollusciciding. Then, the goal of eliminating the transmission of schistosomiasis has been reached in 2004. Sixteen years later, no indigenous cases were detected in Morocco, and only 25 residual cases (resulting from bilharziasis previously treated) are detected, such as in Tata ( 40%), Errachidia (16%), and (12%) in Marrackesh. Similarly, recent national studies conducted on children and the snail reservoir hosts have indicated that no human and molluscs are currently infected with *Schistosoma haematobium*. Actually, timely investigation and management of imported cases has been implemented to prevent the reintroduction of the disease. The Ministry of Health is planning to implement final confirmatory surveys before requesting WHO to proceed with the formal verification process.

## 1. Introduction

Schistosomiasis or bilharzia is the second most common parasitic disease worldwide after malaria [1] caused by trematode worms belonging to the genus *Schistosoma*. It is transmitted by specific freshwater gastropods that are the intermediate hosts of the parasite [2]. Nearly 200 million people in 52 countries are infected, and 80–90% of them live in Africa. Schistosomiasis is responsible for 500,000 deaths per year [3]. In Morocco, bilharziasis due to *Schistosoma haematobium* has existed for several decades. The launching of the national schistosomiasis control program in 1982 had short-term objectives, such as the control of morbidity, infection and transmission, the fight against intermediate hosts, and the reduction of the disease prevalence in foci of the country. While the long-term aim was to eliminate the disease transmission, human treatment was selective and mass treatment was rarely used, only in localities where prevalence exceeded 4% [4–6]. Until the introduction of praziquantel in 1987, niridazole and metrifonate were used for chemotherapy. Snail control campaigns were carried out in focal areas every 1 to 2 months. Along with treatment programs, Morocco targeted the intermediate host of the schistosomes. Control programs recommended environmental modification to eliminate snail habitats, but teams also used the molluscicide (niclosamide) as a form of snail control [4].

The program resulted in a decrease in the number of cases between 1994 and 2003 [7], and the prevalence reached zero indigenous cases in 2004. This study aims to describe the epidemiological profile of urinary schistosomiasis, make a distribution map of the disease in Morocco, and discuss the different stages of the evolution of this disease in Morocco since 1960 until present.

## 2. Methodology

The study is a retrospective descriptive study of schistosomiasis cases recorded during 1960–2018, from published and not published data, such as disease case registers, provincial reports quarterly, action plans, and annual reports, collected from the Direction of Epidemiology and Disease Control of the Ministry of Health, and National Institute of Hygiene, Morocco. In addition, search engines (Google scholar, PubMed etc.) were electronically searched to identify original studies containing the rate of schistosomiasis epidemiology since 1960 until now. The data were entered in Excel software (Excel 2007) and analyzed using SPSS 20.0, and the sectorial geography of cases was done by ArcGIS.

## 3. Results and Discussion

### 3.1. Evolution of Schistosomiasis before the Establishment of National Schistosomiasis Control Program in Morocco (NSCP), 1960–1981

**Figure 1:**
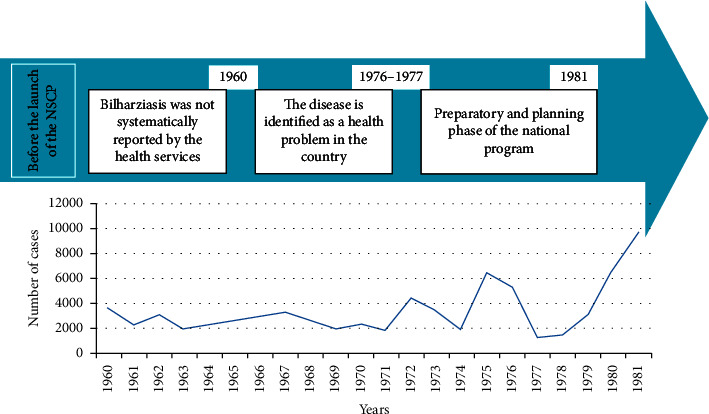
Evolution of schistosomiasis before the establishment of the national schistosomiasis control program.

The appearance of urinary schistosomiasis, caused by *S. haematobium* and transmitted by *B. truncatus*, in Morocco, dates back to the 14^th^ century among caravaneers crossing the Sahara from Timbuktu (Mali) to Tafilalt, who were suffering from “bloodshed” [8]. The history of schistosomiasis in Morocco has been marked by several stages, in 1915. Job, a French military doctor, published for the first time the analysis of some cases of *S. haematobium* contracted by European soldiers in Marrakech in 1914 [9]. The transmission of schistosomiasis increased following development of the modern open-air irrigation network, which, since 1967, caused a rapid spread of the disease to previously unaffected areas [10–13]. Moreover, before 1960, cases of bilharziasis were not systematically reported by the health services. The assessment of the situation was based on indications of infestation reported by the publications of various authors during occasional surveys [14].  Figure 1 summarizes the chronology of the main events that marked bilharzia in Morocco, from 1960 until 1976. The cases of bilharziasis were reported either on the basis of the results of parasitological examination of the urine or on the observation during consultation of free hematuria in adults or children (Figure 1). About 6300 cases were notified in 1975, and between 1977 and 1981, the severity of schistosomiasis increased with increasing egg excretion and transmission (22,010 cases). Indeed, schistosomiasis has posed a serious threat to the country, as it was estimated that 11% of the rural population in 16 of 47 provinces were at risk [15]. For this reason, the Ministry of Public Health has developed a national schistosomiasis control program (this is the preparatory and planning phase of the national program) (Figure 1).

### 3.2. Evolution of Schistosomiasis after Development of the NSCP (1982–2018)

The national schistosomiasis control program was gradually introduced in endemic provinces from 1982 onwards. The program was essentially based on the detection of positive cases and their treatment with niridazole and metrifonate (1982–1986), then praziquantel from 1986; diagnosis by the method of sedimentation of the urine; and the development of prospecting techniques and the organization of malacological surveillance. In addition, there are other strategies of the program, such as sterilizing the human focus, preventing infestation and preventing the development of clinical forms, and finally, mitigating the social and economic consequences of the disease. From 1982, when the program was launched in its operational phase, the number of cases declined from 6580 to in 1993 with a peak of 10.653 cases in 1983. The use of praziquantel as a treatment in 1986 was a limiting factor in the decrease the number of bilharziasis cases in the following years [10–12].

**Figure 2:**
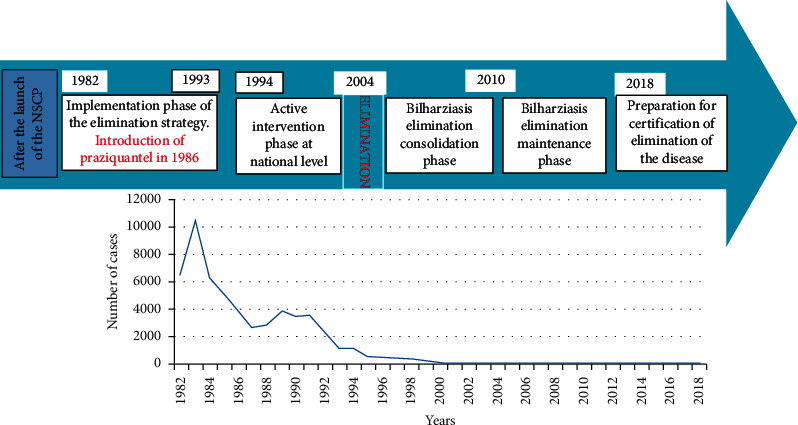
Evolution of schistosomiasis after development of the NSCP.

About 52,470 cases were reported in the phase of implementation of the strategy of elimination 1982–1993 and 3515 during the phase of active intervention in the national level 1994–2003. The results were encouraging according to the recommendations of the national experts and the WHO (Figure 2). The period from 1994 to 2004 was characterized by the strengthening of screening activities in at-risk provinces and by the organization of mass chemotherapy operations at the localities where the incidence of this disease was high [13]. As a result, the epidemiological situation was monitored in the majority of households, and the number of cases dropped from 1108 in 1994 to only 8 cases in 2004. Thus, transmission was stopped in all active foci (Figure 2).

This cessation of transmission was consolidated during the period 2005–2010 by appropriate monitoring activities in all risk areas. Thus, no active transmission has been detected. The National Serological Survey using HAMA EITB and molecular malacological surveys using DraI PCR and SmSl PCR conducted since 2009 has confirmed the interruption of transmission of urinary schistosomiasis in Morocco [4, 5]. A pilot study carried out in 2017 in the region of Tata indicates that some individuals (last remaining cases) are still harboring *Schistosoma* worms which apparently are not shedding eggs; this demonstrates the need for a high sensitivity worm-antigen test as the UCP-LF CAA test. To prevent reemergence of schistosomiasis, the national survey should focus on immigrants, travelers, and all potential risk groups (for example, children and professionally exposed individuals as canal cleaners, car washers, and fishermen) directly with UCP-LF CAA independent of their antibody test result [16].

### 3.3. Geographical Distribution of Bilharziasis Cases

**Figure 3:**
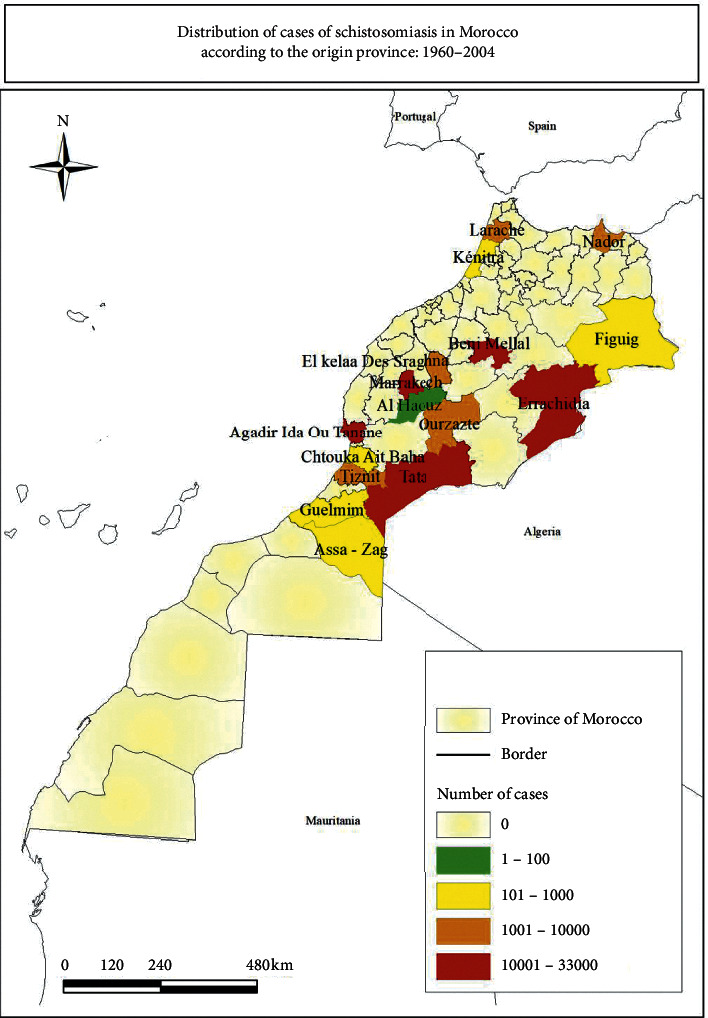
Distribution of cases of schistosomiasis in Morocco according to the origin province: 1960–2018.

Between 1960 and 2004, 129,526 cases were recorded in Morocco. A majority of cases have been recorded in the cities of Agadir (25%), Errachidia (18%), Beni Mellal (13%), Tata (10%), Ouarzazate (7%), El Kelaa des Sraghna (6%), and Marrakesh (6%) (Figure 3). The centralization of these cases in these areas is due to the physicochemical and environmental factors recorded in these provinces, who are favorable for the transmission and the development of the disease, such as irrigation which is the only method of cultivation, the oases of the valleys of major rivers, construction of dams and development of permanent irrigation system, and population movements [12, 13]. In addition, the population is still living near water collections. These provinces with centers of transmission are formed before 1972, and some mass parasitological surveys carried out between 1966 and 1970 in these provinces have revealed average infestation rates varying between 4.5% in households in Marrakesh and 29.0% in Agadir [17]. These provinces were the last areas that declared the elimination of this disease.

### 3.4. Residual Cases: 2004–2018

After the elimination of the disease in 2004, only a few residual sporadic and imported cases [18] from abroad are detected each year.

**Figure 4:**
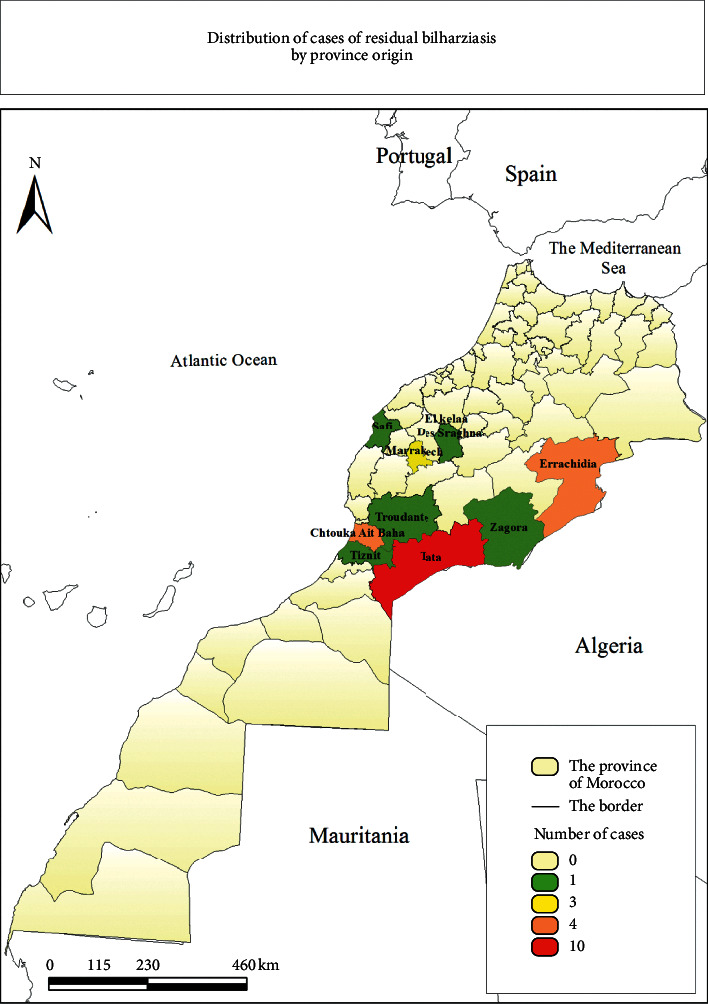
Distribution of residual cases of bilharziasis in Morocco: 2004–2018.

Since 2004 until 2018, 25 residual cases have been detected and recorded, according to the annual reports of the Ministry of Health. According to our retrospective study, 40% of them came from Tata, 16% from Errachidia , 12% from Marrakech, and other provinces (El Kelaa des Sraghna, Chtouka Ait Baha, Taroudant etc.) (Figure 4). These provinces were the last regions that declared the elimination of this disease. These cases are infected with the parasite in the past but are poorly treated because of the problems encountered with the treatment, especially since praziquantel has limits, or the poor drug absorption [16].

### 3.5. Snail Control

According to WHO, snail control will complement mass treatment campaigns and sustain the public health impact to achieve the target of eliminating schistosomiasis as a public health problem [19]. So, the control of molluscs, schistosomiasis intermediate host, has a primary effect on the interruption of transmission. In Morocco, the national schistosomiasis control program neutralizes the mollusc biotope by mechanical, biological, and chemical actions, in order to prevent the development of the parasite. Regarding mechanical control, three techniques have been implicated: modification of the biotope [20, 21], brushing of the edges of sumps after each irrigation [22], and periodic maintenance of the various network structures [23]. In addition, biological control, based on the use of predators (ducks, fish, and parasites), decreases the mollusc proliferation [24]. On the other hand, the niclosamide presents the choice molluscicide for the chemical control of the intermediate host of the parasite [25]. Malacological surveys carried out each year by specialists confirm the absence of *Bulinus truncatus* in most foci. The same context, a molecular study carried out in 2009, showed that none of the snails collected were infected with *S. haematobium* in all the historical endemic areas [5].

### 3.6. Other Successful Control Programs to Eliminate the Disease in Several Areas of the World

The elimination of a parasitic disease is characterized by the interruption of local transmission (reduction to zero of the incidence of indigenous cases) of a specified parasite in a defined geographical area [26]. During the last 20 years, significant progress in the fight has been made following the strategies recommended by WHO. Several countries have reduced the endemicity of schistosomiasis to a low level. However, Morocco has developed, implemented, and managed a process of elimination of the disease through active detection of cases, mass treatment, control of freshwater molluscs, educational and health measures, and intersectoral collaboration. The Brazilian schistosomiasis control programme was planned in the 1970s to prevent the disease [27] which reduced the prevalence of *S. mansoni* from 23% to 6% between 1977 and 2005. The country has managed to eliminate schistosomiasis in five states among of 14 states of the country in Brazil [28]. In Saudi Arabia, *S. haematobium* and *S. mansoni* were endemic in 12 regions [29], and in 2005, a national programme for elimination of schistosomiasis was initiated, which eliminated the disease in several regions in 2007. The schistosomiasis control programme in Tunisia, started in 1970 [28], eliminated schistosomiasis, and the last autochthonous cases occurred in 1981–1982 [28]. In Egypt, the national schistosomiasis control programme (NSCP) started in 1976 [29]. Since the late 1980s to 2007, the prevalence of *S. mansoni* and *S. haematobium* in 2006 and the overall prevalence in Egypt dropped to <3% [29]. In the Islamic Republic of Iran, the control of urinary schistosomiasis was started in 1959 [30], and the prevalence of *S. haematobium* infection declined to 0.7% in 1979 [31]. In 1950, the Japanese government launched a campaign to combat parasitic diseases, which leads to the reduction the prevalence of *S. japonicum*. Indeed, in 1977, schistosomiasis was declared eliminated [32, 33].

In Morocco, even if the last indigenous case was reported in 2004, the detection of a number of imported and residual cases each year as well as the presence of the intermediate host constitutes a risk of reintroduction of the disease. Therefore, a possible resumption of the transmission of schistosomiasis in Morocco should be considered with great attention as there are migration, blocks, travelers, lack of rapid test at airports etc. Additional research to address gaps, develop tools, and optimize the impact of existing programmes with snail control will complement the core strategic approach [19].

## 4. Conclusion

Thanks to control activities within the national schistosomiasis control program for more than three decades, the goal of eliminating the transmission of schistosomiasis has been reached. Therefore, possible resumption of transmission of schistosomiasis in Morocco should be monitored with great attention.

## Data Availability

All data are cited in the manuscript.
